# *cryptochrome* genes form an oscillatory loop independent of the *per*/*tim* loop in the circadian clockwork of the cricket *Gryllus bimaculatus*

**DOI:** 10.1186/s40851-017-0066-7

**Published:** 2017-04-06

**Authors:** Atsushi Tokuoka, Taichi Q. Itoh, Shinryo Hori, Outa Uryu, Yoshiki Danbara, Motoki Nose, Tetsuya Bando, Teiichi Tanimura, Kenji Tomioka

**Affiliations:** 1 Graduate School of Natural Science and Technology, Okayama University, 3-1-1 Tsushima-naka, Kita-ku, Okayama, 700-8530 Japan; 2 Graduate School of Science, Kyushu University, Fukuoka, 819-0395 Japan; 3 Okayama University Graduate School of Medicine, Dentistry and Pharmaceutical Sciences, Okayama, 700-8558 Japan

**Keywords:** Circadian clock, Clock gene, *Cryptochrome*, Insect, Molecular oscillatory mechanism

## Abstract

**Background:**

Animals exhibit circadian rhythms with a period of approximately 24 h in various physiological functions, including locomotor activity. This rhythm is controlled by an endogenous oscillatory mechanism, or circadian clock, which consists of cyclically expressed clock genes and their product proteins. *cryptochrome* (*cry*) genes are thought to be involved in the clock mechanism, and their functions have been examined extensively in holometabolous insects, but in hemimetabolous insects their role is less well understood.

**Results:**

In the present study, the role of *cry* genes was investigated using RNAi technology in a hemimetabolous insect, the cricket *Gryllus bimaculatus*. Using a molecular cloning approach, we obtained cDNAs for two *cry* genes: *Drosophila*-type *cry1* (*Gb’cry1*) and mammalian-type *cry2* (*Gb’cry2*). *Gb’cry2* has six splicing variants, most of which showed rhythmic mRNA expression. *Gb’cry1* ^RNAi^ treatment had only a limited effect at the behavioral and molecular levels, while *Gb’cry2* ^RNAi^ had a significant effect on behavioral rhythms and molecular oscillatory machinery, alone or in combination with *Gb’cry1* ^RNAi^. In *Gb’cry1*/*Gb’cry2* double-RNAi crickets, most clock genes showed arrhythmic expression, except for *timeless*, which retained clear rhythmic expression. Molecular analysis revealed that some combination of *Gb’cry1* and *Gb’cry2* variants suppressed CLK/CYC transcriptional activity in cultured cells.

**Conclusion:**

Based on these results, we propose a new model of the cricket’s circadian clock, including a molecular oscillatory loop for *Gb’cry2*, which can operate independent of the *Gb’per*/*Gb’tim* loop.

## Background

Circadian rhythms are a biological rhythm with a period of approximately 24 h, which can be observed in various physiological functions of animals, including general activity, hormonal secretion, and metabolism. Rhythmicity is generated by an endogenous mechanism, the so-called circadian clock, which consists of cyclic expression of clock genes and their product proteins. In insects, the core clock components are *period* (*per*), *timeless* (*tim*), *Clock* (*Clk*), and *cycle* (*cyc*) [1, 2]. *Clk* and *cyc* encode the transcription factors CLOCK (CLK) and CYCLE (CYC), which form a heterodimer and activate transcription of *per* and *tim*. Transcripts of *per* and *tim* are subsequently translated to the product proteins PERIOD (PER) and TIMELESS (TIM), which heterodimerize, enter the nucleus, and inhibit transcriptional activity of CLK/CYC. This negative feedback loop produces the 24 h oscillation. The two *cryptochrome*s, *cry1* (or *Drosophila*-type *cry*) and *cry2* (or mammalian-type *cry*), are also important components involved in the insect clock. *cry* proteins are members of the photolyase family, and *cry1* functions as a photoreceptor, resetting the clock through TIM degradation upon exposure to blue light in *Drosophila* and some other insects [3, 4]. *cry2* is thought to be a component of the clock, working together with PER to form a feedback machinery [5]. This hypothesis has been supported by a reporter assay using cultured cells [3, 6]. However, to date the roles of *cry* genes have been investigated mostly in holometabolous insects, meaning their functions in hemimetabolous insects must be investigated if we are to understand their role in these insects and their functional diversification.

The cricket, *Gryllus bimaculatus*, is well suited to the study of these questions, since the molecular machinery of the clock has been extensively studied and RNAi can be used to dissect the roles of clock genes [7, 8]. The clock is located in the optic lobe, and its oscillatory components include *Gb’per*, *Gb’tim*, *Gb’Clk*, and *Gb’cyc*, as in *Drosophila*, but their roles differ substantially from those in *Drosophila* [7]. Similar to the case in *Drosophila*, *Gb’per* and *Gb’tim* are rhythmically expressed and their transcripts increase at night, peaking from early night to midnight [9, 10]. However, unlike in *Drosophila*, *Gb’cyc,* not *Gb’Clk*, is rhythmically expressed [11, 12]. Double-stranded RNA (dsRNA) treatment of *Gb’per* and *Gb’Clk* abolished the locomotor rhythm and the molecular expression rhythms of *Gb’per* and *Gb’tim* [9, 11, 13]. However, *Gb’tim* and *Gb’cyc* RNAi arrests the rhythmic expression of *Gb’per* and *Gb’tim*, but does not prevent behavioral rhythmicity [10, 12], suggesting the existence of other oscillatory components. We suggest that *cry* is a candidate component as, in some insects, CRY functions as a transcriptional repressor by itself, at least in cultured cells [3].

In the present study, we obtained two *cry* genes in *G. bimaculatus* by molecular cloning and confirmed that they are homologous to *cry1* (*Gb’cry1*) and *cry2* (*Gb’cry2*) by structural analysis. PCR analysis revealed that there are six splicing variants of *Gb’cry2*. We then examined their expression profiles and functional roles in the clock machinery by RNAi experiments. RNAi of *Gb’cry2* substantially changed the free-running period of the locomotor rhythm, and the change was further enhanced by *Gb’cry1* ^RNAi^, suggesting that the *cry* genes may be involved in the determination of the free-running period. Although the molecular oscillation of clock genes was mostly halted by *Gb’cry1/Gb’cry2* double RNAi, *Gb’tim* retained its rhythmic expression. Cellular reporter assays revealed that some combination of *Gb’cry1* and *Gb’cry2* variants represses CLK/CYC transcriptional activity. We propose a unique model for the molecular clock of the cricket that incorporates these new findings and previously described properties of clock genes.

## Methods

### Experimental animals

Third instar nymphs and adult male crickets, *Gryllus bimaculatus*, were used. These were purchased or obtained from a laboratory colony maintained under standard environmental conditions with a lighting regimen of LD 12:12 (light: 0600-1800; Japan Standard Time) and a constant temperature of 25 ± 1.0 °C. They were fed laboratory chow and water.

### Cloning and structural analysis of the *cry* genes

Total RNA was extracted with ISOGEN (Nippon Gene, Tokyo, Japan) from 20 heads of third instar nymphs collected at ZT 10 (ZT stands for zeitgeber time and ZT0 corresponds to lights-on and ZT12 to lights-off). We used 4.5 μg of total RNA for reverse transcription to obtain cDNA, using SuperScript II (Invitrogen, Carlsbad, CA, USA). Using the single-stranded cDNA as a template, we performed PCR with degenerate primers deduced from the conserved amino acid sequences among insect *Drosophila*-type *cryptochrome* (*cry1*) and mammalian-type *cryptochrome* (*cry2*) homologues. The primers used for *cry1* and *cry2* were 5′-CAGGAACAAAGATGGTGGGATAYAAYMGNATG-3′ for forward and 5′-CCAGTTTCCTGCGCACACNSWCCARTC-3′ for reverse, and 5′-TGTTCGTGATCCGAGGACARCCNGCNGA-3′ for forward and 5′-CACTGGGATGTACTTTCGGATGWARTCNCCRTT-3′ for reverse, respectively. The PCR conditions employed were 30 s for denaturation at 95 °C, 30 s for annealing at 57 °C, and 1 min 30 s for extension at 72 °C for 35 cycles with ExTaq DNA polymerase (Takara, Otsu, Japan). The purified fragment was cloned into TOPO-pCR II vector (Invitrogen) and sequenced with BigDye Terminator v3.1 Cycle Sequencing Kit (Applied Biosystems, Foster City, CA, USA). 5′ and 3′ RACEs for *cry1* and *cry2* were done with GeneRacer kit (Invitrogen) and SMARTer RACE cDNA Amplification kit (Takara) with gene specific primers, 5′-TGGTTCACAATCCTGCTCAA-3′ and 5′-ATGGAGCGCAATCCTATCTG-3′, and 5′-CCACTTGGCTAAGGCTTCTG-3′ and 5′-GCAGTTCCATCAAGTCAGCA-3′, respectively. RACE fragments were purified, cloned and sequenced as mentioned above. Sequences were analyzed by Genetyx ver. 6 (Genetic Information Processing Software, Tokyo, Japan) and BioEdit ver. 7.0.5 (Biological Sequence Alignment Editor, Ibis Therapeutic, Carlsbad, CA, USA). There were six splicing variants in the cricket *cry2* cDNA. For cloning these cDNAs, primer sets of 5′-ATGCAGGTACCATGGGAAGCACACTTGCATT-3′ for forward and 5′-ATGCAGCGGCCGCAATTGTGCTGTTGATTTAAAC-3′ for reverse, or 5′-ATGCAGGTACCAGTGCTCGTGTGTGTGTTTG-3′ for forward and 5′-ATGCAGCGGCCGCAATTGTGCTGTTGATTTAAAC-3′ for reverse were used. Exon/intron structure of *cry* genes were analyzed with genomic DNA sequence data.

Amino acid sequences of CRY1 and CRY2 were aligned using ClustalW in MEGA 7.0. A phylogenetic tree of CRYs was constructed using maximum likelihood methods based on JTT matrix-based model in MEGA 7.0. Sequences of known insect *cry1* and *cry2* genes were obtained from GenBank.

### Measurement of mRNA levels

Quantitative real-time reverse transcription polymerase chain reaction (qPCR) and reverse transcription polymerase chain reaction (RT-PCR) were used to measure mRNA levels. Total RNA was extracted and purified from six adult male optic lobes with TRIzol Reagent (Invitrogen). To remove contaminating genomic DNA, the total RNA was treated with DNase I. About 500 ng total RNA of each sample was reverse transcribed with random hexamers using PrimeScript RT reagent Kit (Takara). Real-time PCR was performed by Mx3000P Real-Time PCR System (Stratagene, La Jolla, CA, USA) using FastStart Universal SYBR Green Master (Roche, Tokyo, Japan) including SYBR Green with primers designed for *Gb’cry1*, *Gb’cry2*, *Gb’per* (BAG48878), *Gb’tim* (BAJ16356), and *Gb’rpl18a* (DC448653) (Table 1). In all cases, a single expected amplicon was confirmed by melting analysis. Quantification was performed based on a standard curve obtained from a known amount of templates. The results were analyzed using the software associated with the instrument.

**Table 1:** Primers used for qPCR, PCR, dsRNA synthesis, and cloning of coding regions for cell-based assay

Primers	Forward	Reverse
For qPCR		
*Gb’cry1*	5′-AAAGCCAGTTACGCTGATGG-3′	5′-TGTAATCTCAGGCCATGTCG-3′
*Gb’cry2*	5′-AGCACCATCACACACTTCACA-3′	5′-ACACTCAGCGCAATCCACAC-3′
*Gb’per*	5′-AAGCAAGCAAGCATCCTCAT-3′	5′-CTGAGAAAGGAGGCCACAAG-3′
*Gb’tim*	5′-CCAAAGACAGGAAGCAGACC-3′	5′-GAATCCCAACACCAAAATGG-3′
*Gb’rpl18a*	5′-GCTCCGGATTACATCGTTGC-3′	5′-GCCAAATGCCGAAGTTCTTG-3′
For PCR		
*Gb’cry2-x2a*	5′-GTGGTATCAACGCAGAAGTA-3′	5′-TCTAGGCACTGTAGTAGGAA-3′
*Gb’cry2-x4*	5′-TTCCGATGCATTTTCATCC-3′	5′-CGCCAAAGCTTCTGATTCTCC-3′
*Gb’cry2-x10b*	5′-CTCCTGAGAGTGTGCAACGT-3′	5′-ACTTGATGGAACTGCTGCCA-3′
For dsRNA synthesis		
*Gb’cry1#d1*	5′-TAATACGACTCACTATAGGGGTTTCGACATGGCCTGAGAT-3′	5′-AATTAACCCTCACTAAAGGGCTTCTGGCGTTGGAAAATGT-3′
*Gb’cry1#d2*	5′-TAATACGACTCACTATAGGGTGGGAACAAGGAAAGACTGG-3′	5′-AATTAACCCTCACTAAAGGGCGACAATGAGGTGGAGGATT-3′
*Gb’cry2#d1*	5′-TAATACGACTCACTATAGGGCTGCGACAAATAACCCCAAC-3′	5′-AATTAACCCTCACTAAAGGGCTCTCAGGAGCATTCCAAGG-3′
*Gb’cry2#d2*	5′-TAATACGACTCACTATAGGGAAGCACACTGTGCATTGGTT-3′	5′-AATTAACCCTCACTAAAGGGCCGTTCTTTTCGATGATGCT-3′
*Gb’per*	5′-TAATACGACTCACTATAGGGCATTCATCGACTTCGTTCACC -3′	5′-AATTAACCCTCACTAAAGGGCTGAACGCCCAATCATGTCT -3′
*Gb’tim*	5′-AATTAACCCTCACTAAAGGGGTAAAGAAGATAGAGAGTAT-3′	5′-AATTAACCCTCACTAAAGGGTTGGAGAGAACTGAAGAGGT-3′
*Gb’Clk*	5′-TAATACGACTCACTATAGGGTACATCACGCCATAGCCTTG -3′	5′-AATTAACCCTCACTAAAGGGGGGATTGCTCTTCTTTGCTG -3′
*Gb’cyc*	5′-TAATACGACTCACTATAGGGCGTGCACTCGTACACTGAGG-3′	5′-AATTAACCCTCACTAAAGGGAGGTTCTGCTGCTTCTTTCG-3′
*DsRed2*	5’-TAATACGACTCACTATAGGGTCATCACCGAGTTCATGCG-3′	5′-TAATACGACTCACTATAGGGCTACAGGAACAGGTGGTGGC-3′
For cloning of coding region for cell-based assay		
*Gb’cry1*	5′-ATGCAGGTACCAAGCCAGTTACGCTGATGGT-3′	5′-GGGCTCGAGAGTGGTCAAAGCAATTATCAT-3′
*Gb’cry2#p1*	5′-ATGCAGGTACCATGGGAAGCACACTTGCATT-3′	5′-ATGCAGCGGCCGCAATTGTGCTGTTGATTTAAAC-3′
*Gb’cry2#p2*	5′-ATGCAGGTACCAGTGCTCGTGTGTGTGTTTG-3′	5′-ATGCAGCGGCCGCAATTGTGCTGTTGATTTAAAC-3′

*Gb’cry1, Gb’cry2#p1*, *Gb’cry2#p2* were used for cloning of *Gb’cry1*, *Gb’cry2*a ~ c, and *Gb’cry2*d ~ f, respectively

To estimate expression levels of *Gb’cry2* variants, semi-quantitative RT-PCR was performed with *Gb’cry2-x2a*, *Gb’cry2-x4* and *Gb’cry2-x10b* primer sets that were designed to detect variants with or without *Gb’cry2* exon2a, 4 and 10b, respectively (Table 1). *Gb’rpl18a* was also used as internal reference. The PCR conditions were 10 s for denaturation at 98 °C, 30 s for annealing at 52 °C, and 30 s for extension at 72 °C for 35 cycles with Emeraldamp® MAX PCR Master Mix (Takara, Otsu, Japan) for *Gb’cry2* exon2a, 10 s at 98 °C, 30 s at 55 °C, and 30 s at 68 °C for 33 cycles with KOD FX Neo (Toyobo, Osaka, Japan) for exon4 and 10b, 10 s at 98 °C, 30 s at 55 °C, and 30 s at 72 °C for 28 cycles with Emeraldamp® MAX PCR Master Mix for *Gb’rpl18a.* The PCR samples were electrophoresed on 1.5% agarose gels in TBE buffer (89 mM Tris-base pH 7.6, 89 mM boric acid, 2 mM EDTA). Gels were stained with GelRed^TM^ nucleic acid gel stains (Biotium, CA, USA) and photographed on a 280 nm UV light box (TP-20MP, ATTO, Tokyo, Japan). The gel images were digital-imaged and quantified by imageJ software (available from https://imagej.nih.gov/ij/). The quantity of the PCR samples were estimated relative to the internal reference gene, *Gb’rpl18a*, amplified with the same PCR protocol.

For both qPCR and RT-PCR, the values were normalized with those of *Gb’rpl18a* at each time point. Results of 3–5 independent experiments were used to calculate the mean ± SEM.

### RNAi

dsRNA for *Gb’cry1*, *Gb’cry2*, *Gb’per* (GenBank/EMBL/DDBJ Accession No. BAG48878), *Gb’tim* (BAJ16356), *Gb’Clk* (AB738083), *Gb’cyc* (AB762416) and *DsRed2* derived from a coral species (*Discosoma* sp.), were synthesized using MEGAscript High Yield Transcription kit (Ambion, Austin, TX, USA). For *Gb’cry1* and *Gb’cry2*, cDNAs prepared as described above, were used as templates of PCR, which was performed with ExTaq DNA polymerase (Takara). The T7 or T3 containing primers used were listed in Table 1. Amplified *Gb’cry1#d1* (575 bp), *Gb’cry1#d2* (515 bp), *Gb’cry2#d1* (463 bp), *Gb’cry2#d2* (423 bp), *Gb’per* (456 bp), *Gb’tim* (519 bp), *Gb’Clk* (407 bp), and *Gb’cyc* (450 bp) fragments were extracted with phenol/chloroform and precipitated with ethanol then resuspended in Ultra Pure Water (Invitrogen). For *DsRed2* dsRNA, linearized *DsRed2* fragment was amplified from pDsRed2-N1 (Clontech, Mountain View, CA, USA), with the primers shown in Table 1. With each of these linearized fragments as a template, RNA was synthesized with T7 or T3 RNA polymerase. Synthesized RNAs were extracted with phenol/chloroform, and suspended in 50 μl TE solution after isopropanol precipitation. The yield and quality of RNA were assessed by absorbance using a spectral photometer (Genequant Pro, Amersham Bioscience, Piscataway, NJ, USA) and the same amount of sense and antisense RNA were mixed. The RNAs were denatured for 5 min at 100 °C and annealed by a gradual cool down to room temperature. After ethanol precipitation, the obtained dsRNA was suspended in Ultra Pure Water (Invitrogen) and adjusted to the final concentration of 20 μM. The dsRNA solution was stored at –80 °C until use. 760 nl of dsRNA solution was injected with a nanoliter injector (WPI, Sarasota, FL, USA) into the abdomen of adults anesthetized with CO_2_.

### Assays in cultured cells

The coding sequences of *cry* genes, *Gb’cry1* and *Gb’cry2*a–f, were subcloned individually into *pAc5.1B-V5/His* (Invitrogen, Carlsbad, CA). Primers used for cloning are listed in Table 1. Cultured *Drosophila* S2 cells were plated in 24-well tissue culture plates in Shields and Sang M3 insect medium (Sigma, St Louis, MO) supplemented with 12.5% fetal bovine serum (Biowest, Canada) and antibiotics (12.5 U/ml penicillin, 12.5 mg/ml streptomycin; GIBCO, Grand Island, NY). S2 cells were transfected by Effectene Transfection Reagent (Qiagen, Hilden, Germany) with 100 ng of *pAct-Clk* as an activator [14], 100 ng of *Drosophila tim-luc* [14], which contains a typical E-box [15] showing a relatively high expression [16], and 10 ng of *pAc5.1-Rluc* [17] as a positive control for luciferase activity, along with a combination of 500 ng of *pAc5.1-Gb’cry1* and *pAc5.1-Gb’cry2*a ~ f. As needed, the empty vector *pAc5.1B-V5/His* was used instead of the vector constructs of *Gb’cry1* and *Gb’cry2* genes to ensure that an equal amount of DNA was used for transfection in each well. Forty-eight hours after transfection, luciferase assays of transfected cells were carried out using the Dual-Luciferase® Reporter Assay System (Promega Corporation, Madison, Wisconsin, USA) and then normalized to *Rluc* activity as a control for transfection efficiency. Assays were performed at least three times.

### Behavioral analysis

Locomotor activities were recorded in the same way as described previously [9]. Briefly, the final instar nymphs or adult crickets were individually housed in a transparent plastic box (18 × 9 × 4.5 cm) with a rocking substratum. The number of substratum rocking was recorded every 6 min by a computerized system. Food and water were provided *ad libitum*. The actographs were placed in an incubator (MIR-153, Sanyo Biomedica, Osaka, Japan) in which temperature was kept at 25 ± 0.5 °C and lighting conditions were maintained by a cool white fluorescent lamp connected to an electric timer. The light intensity was 600–1000 lux at the animal’s level, varying with the proximity to the lamp. The raw data were displayed as conventional double-plotted actograms to judge activity patterns, and the free-running period was calculated by the chi-square periodogram [18] with Actogram J (freely available at http://actogramj.neurofly.de/) [19]. If a peak in the periodogram appeared above the 0.05 confidence level (alpha = 0.005), the power value (height of the peak above the confidence level) was greater than or equal to 10, and the width of the peak was greater than or equal to 2, the period for the peak was designated statistically significant [20].

### Statistical analysis

The one-way analysis of variance (ANOVA) followed by a post hoc Tukey-test was used to compare the differences in means of mRNA levels between the different time points and the differences in means of free-running periods and phase angle differences between insect groups with different treatments. To compare the means of two groups, *t*-test was used. Dunnett’s-test was also used for multiple comparisons, where applicable. Significance of the daily and circadian rhythmicity in mRNA expression was examined by the single cosinor method [21], fitting a cosine curve of 24 h period using Time Series Analysis Serial Cosinor 6.3 (Expert Soft Technologie, Richelieu, France). In all statistical tests, the significance level was set at *P* < 0.05.

## Results

### Cloning and structural analysis of *Gb’cry1* and *Gb’cry2*

**Fig. 1:**
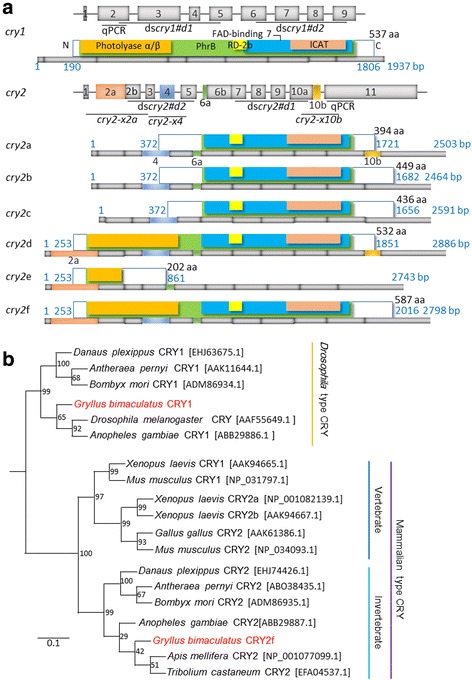
Sequence alignments and a phylogenetic tree of *Gb’cry1* and *Gb’cry2*. **a** Schematic structure of *Gb’cry1* and *Gb’cry2* cDNAs and their deduced product proteins, showing conserved domains, photolyase α/β (*orange*), PhrB (*green*), RD-2b (*yellow*), FAD-binding 7 (*blue*), and inhibition of CLOCK-ARNTL-mediated transcription (ICAT) domain (*pink*). At the top of schemes of each gene exon/intron structure is illustrated. There are six splicing variants for *Gb’*CRY2, of various lengths. The bars under each protein scheme show cDNA structure and the colored portions indicate the respective exons. Numbers shown at the right of protein illustration indicate the number of amino acid residues (aa) and the blue numbers indicate the number of base pairs. The regions used for qPCR and dsRNA synthesis are also shown. *cry2-x2a*, -*x4*, and –*x10b* are for PCR for amplification of variants with or without exon 2a, 4, and 10b, respectively. **b** A phylogenetic tree of known insect and vertebrate CRY proteins. CRY amino acid sequences were analyzed, and the phylogenetic tree was inferred by the maximum likelihood method in MEGA 7.0. The GenBank or RefSeq accession numbers are indicated in brackets. The reference bar indicates the distance as number of amino acid substitutions per site

*cry1* and *cry2* homologues were cloned from the optic lobe of *G. bimaculatus* using a degenerate PCR strategy with degenerate primers based on conserved amino acid sequences from insects *cry1* and *cry2* homologues. A *Gryllus bimaculatus cry1* (*Gb’cry1*) fragment of 1116 bps and a *cry2* (*Gb’cry2*) fragment of 876 bps, including the FAD-binding 7 domain and a part of PhrB, was first obtained. By 3′ rapid amplification of cDNA ends (RACE) and 5′ RACE, we obtained a full length of 1937 bp *Gb’cry1* cDNA (GenBank/EMBL/DDBJ Accession No. LC202047). The *Gb’cry1* cDNA had 189 bp 5′-untranslated regions (UTR) and 131 bp 3′-UTR. Genomic DNA analysis revealed that *Gb’cry1* consists of nine exons, and no splicing variants were detected by RT-PCR. The putative product protein *Gb’*CRY1 consists of 537 amino acid residues (Fig. 1a).

As to *Gb’cry2,* we obtained six variants of cDNAs, those are *Gb’cry2*a ~ f (GenBank/EMBL/DDBJ Accession No. LC202048, LC202049, LC202050, LC202051, LC202052, LC202053) (Fig. 1a). The lengths of cDNA varied among the variants. Their structures are illustrated in Fig. 1a with deduced proteins. With genomic sequence analysis, the *Gb’cry2* gene was shown to consist of 11 exons and the splicing variants were shown to commonly include exons 1–3 and 5–11 with variable lengths of exons 2, 6 and 10. Exceptionally, a variant *Gb’cry2*e lacked exon 4. *Gb’cry2*a ~ c had a short exon 2 lacking the 2a region, *Gb’cry2*c with short exon 6 lacking 6a region, and *Gb’cry2*a, d with longer exon 10 including a 10b region. From these mRNAs six different proteins are deduced. *Gb’*CRY2a ~ c lack the photolyase α/β domain, because the start codon is located in middle of exon 4, *Gb’*CRY2c has a shorter N-terminal region lacking exon 6a region, and *Gb’*CRY2a and -d have a shorter C-terminal region because of stop codon in exon 10b. *Gb’*CRY2e is the smallest lacking most part and consists of only 202 amino acid residues, while *Gb’*CRY2f is the largest, includes all coding regions, and consists of 587 amino acid residues.

A BLAST database search indicated that the amino acid sequence of *Gb’*CRY1 has 55–57% identities along the entire length of the protein with those of CRY1 characterized in other insects, including the fruit fly *Drosophila melanogaster* (GenBank/EMBL/DDBJ accession No. AAF55649.1), the monarch butterfly, *Danaus plexippus* (EHJ63675.1), the silkmoth, *Antheraea pernyi* (AAK11644.1), and the mosquito, *Anopheles gambiae* (ABB29886.1, Table 2). The longest *Gb’*CRY2f has 62–79% identity along the entire length of the protein with those of known insect CRY2s and mouse CRYs, including the monarch butterfly, *D. plexippus* (EHJ74426.1), the silkmoth, *A. pernyi* (ABO38435.1), the honeybee, *Apis mellifera* (NP_001077099.1), the mosquito, *A. gambiae* (ABB29887.1), the red flour beetle, *Tribolium castaneum* (EFA04537.1) and the mouse, *Mus musculus* (CRY1, NP_031797.1, CRY2, NP_034093.1) (Table 2).

**Table 2:** Overall amino acid identity (%) of whole sequence and functional domains of *Gryllus bimaculatus* CRY1 and CRY2f with their insect and mammalian orthologues

Species	Whole sequence	DNA photolyase	FAD binding 7	RD-2b	ICAT	PhrB
*Gb’*CRY1						
*Drosophila melanogaster* CRY	55	59	59	51	63	57
*Danaus plexippus* CRY1	56	56	64	54	69	58
*Antheraea pernyi* CRY1	57	59	63	48	69	59
*Anopheles gambiae* CRY1	56	59	61	51	65	58
*Gb’*CRY2f						
*Danaus plexippus* CRY2	67	82	82	75	81	80
*Antheraea pernyi* CRY2	71	80	85	82	84	81
*Apis mellifera* CRY2	77	85	92	96	91	87
*Anopheles gambiae* CRY2	73	82	91	100	89	85
*Tribolium castaneum* CRY2	79	79	91	96	89	85
*Mus musculus* CRY1	64	65	82	79	79	74
*Mus musculus* CRY2	62	65	79	75	74	71

The deduced proteins *Gb’*CRY1 and *Gb’*CRY2f commonly had five highly conserved regions that are characteristic of CRY proteins (Fig. 1a): (i) DNA photolyase α/β domain, (ii) FAD-binding 7 domain, (iii) RD-2b domain, which is necessary for nuclear localization and the repression of CLOCK: BMAL-mediated transcription, (iv) inhibition of CLOCK-ARNTL-mediated transcription (ICAT) domain, and (v) PhrB domain. Identity of these domains had 51–69% in *Gb’*CRY1, while those in *Gb’*CRY2f had high identity of 52–100% and also share high identity with mouse CRY1 and CRY2 (Table 2). A phylogenetic tree based on the amino acid sequences of CRYs from known insects and some vertebrates revealed that *Gb’*CRY1 and *Gb’*CRY2f form separate clades with CRY1 and CRY2 in other insects (Fig. 1b).

### Expression profiles of *Gb’cry1* and *Gb’cry2* mRNA in the optic lobe

**Fig. 2:**
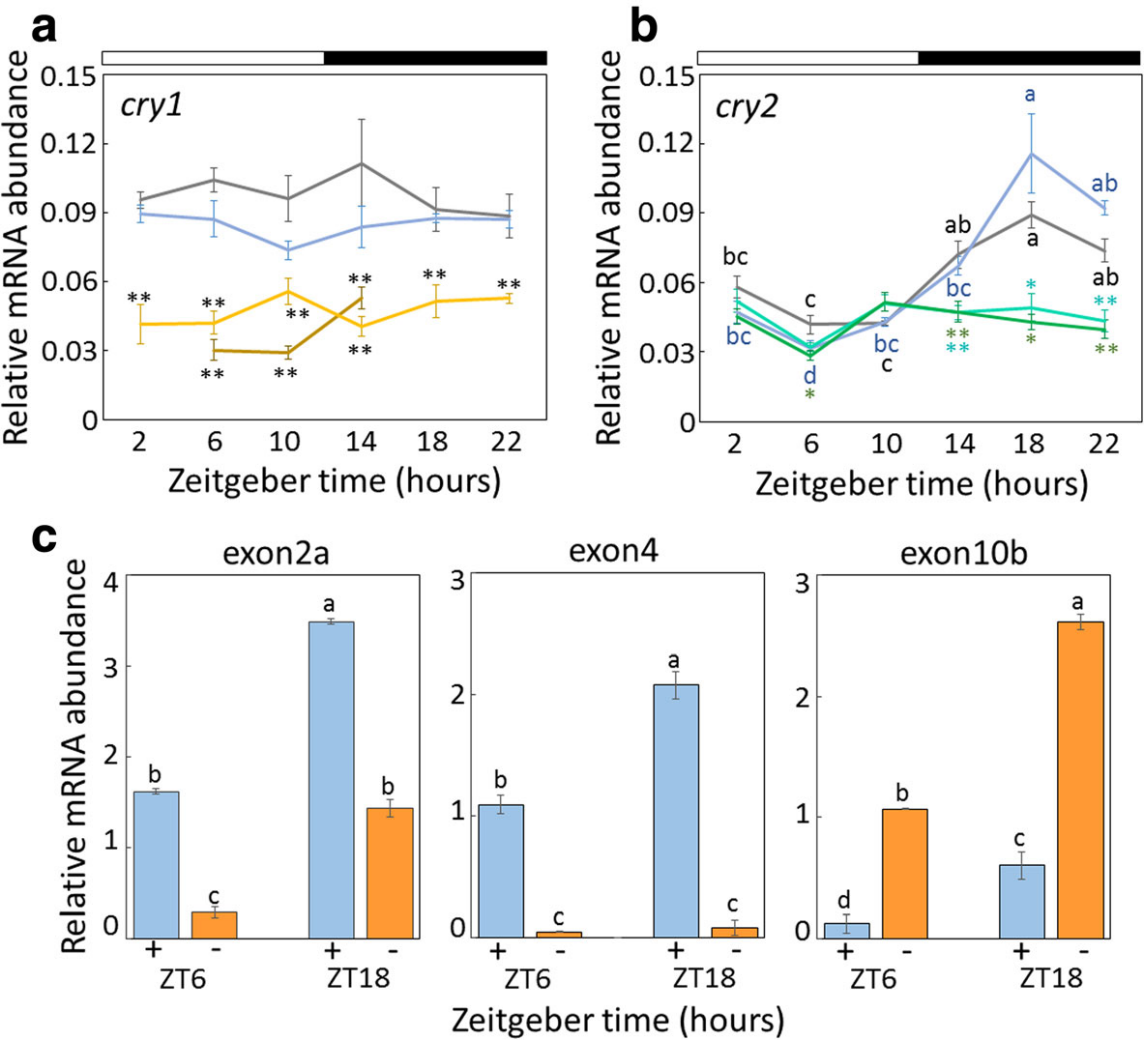
Expression profiles of *Gb’cry1* (**a**) and *Gb’cry2* mRNA (**b**, **c**) in *Gryllus bimaculatus* optic lobes under LD 12:12. In (**a**) and (**b**), abundance of *Gb’cry1* and *Gb’cry2* mRNA is shown for intact (*blue*), ds*DsRed2* (*gray*), ds*Gb’cry1#d1* (*yellow*), ds*Gb’cry1#d2* (*dark yellow*), ds*Gb’cry2#d1* (*dark green*), ds*Gb’cry2#d2* (*green*) treated crickets. Total RNA was extracted from the optic lobes that were collected at 4-hour intervals starting at 2 h after lights-on (ZT 2). *White* and *black bars* indicate *light* and *dark*, respectively. *Gb’cry1* was rather constitutively expressed. *Gb’cry2* showed rhythmic expression in intact and *DsRed2* ^RNAi^ crickets (cosinor, *P* < 0.01), while the rhythm disappeared when treated with ds*Gb’cry2* (cosinor, *P* > 0.05). Note that dsRNA significantly reduced mRNA levels of respective genes, while *DsRed2* dsRNA had no significant effect. *Asterisks* indicate significant difference between control (*DsRed2* ^RNAi^) and treated crickets (*P < 0.05, **P < 0.01, Dunnett’s test). **c** Semi-quantification of *Gb’cry2* mRNA levels at ZT6 and ZT18 with (*blue*) and without (*orange*) exon 2a, 4, or 10b. Different lower case letters indicate that values are significantly different with each other (ANOVA followed by Tukey-test, *P* < 0.05). For (**a**–**c**), data collected from three or five independent experiments were averaged and plotted as mean ± SEM. The abundance of *Gb’rpl18a* mRNA was used as an internal reference

To examine whether *Gb’cry1* and *Gb’cry2* transcript oscillated in the optic lobe, their mRNA levels under LD12:12 were measured using qPCR in the adult male crickets. Primers used for qPCR were shown in Fig. 1a and Table 1. As shown in Fig. 2a, *Gb’cry1* appeared to be constitutively expressed, and no clear daily rhythm was observed (ANOVA, F_5,23_ = 1.03, *P* > 0.42; cosinor, *P* > 0.05). For *Gb’cry2*, rhythmic mRNA expression was found with a peak during the midnight (ZT18, ANOVA, F_5,21_ = 21.95, *P* < 0.01; cosinor, *P* < 0.01) when all the splicing variants were amplified with primers designed at exon 11 (Fig. 2b). The amplitude of the rhythm was approximately 4-fold.

We then examined the expression levels of *Gb’cry2*a ~ c and *Gb’cry2*d ~ f with short and long exon 2 using a primer set *Gb’cry2-x2a* (Table 1), respectively, in the optic lobe at ZT 6 and 18 by a semi-quantitative method, RT-PCR. Both of *Gb’cry2*a ~ c and *Gb’cry2*d ~ f mRNAs with short and long exon 2, respectively, showed a higher level at ZT18, but the expression level was much greater in the latter (Fig. 2c; ANOVA, F_3,11_ = 454.26, *P* < 0.01). When the levels of *Gb’cry2*a,d and *Gb’cry2*b, c, e, f with long and short exon 10, respectively, were examined using a primer set *Gb’cry2-x10b* (Table 1), both were again higher at ZT18 but the levels of *Gb’cry2*b, c, e, f were higher (Fig. 2c; ANOVA, F_3,11_ = 110.3, *P* < 0.01). These results suggest that the *Gb’cry2*f was expressed most abundantly. We also compared the levels of *cry2*e that lacked exon 4 and *Gb’cry2*a ~ d,f that included exon 4 using a primer set *Gb’cry2-x4* (Table 1). The results showed that they again showed higher expression at ZT18 (ANOVA, F_3,11_ = 167.36, *P* < 0.01) and that most of expressed *Gb’cry2* variants were those including exon 4 and the *cry2*e expression was only negligible (Fig. 2c).

### RNAi suppresses *Gb’cry1* and *Gb’cry2* expression

We examined the effects of dsRNA of *Gb’cry1* (ds*Gb’cry1*) and *Gb’cry2* (ds*Gb’cry2*) on respective transcript levels, by measuring their mRNA levels in the optic lobe of the adult male crickets by qPCR. ds*Gb’cry2#d1* and ds*Gb’cry2#d2* were synthesized for the region included in all splicing variants (Fig. 1a). Treatment of ds*Gb’cry1#d1* or ds*Gb’cry1#d2* significantly reduced *Gb’cry1* mRNA levels throughout the day (Fig. 2a). In the crickets injected with ds*Gb’cry2#d1* or ds*Gb’cry2#d2*, the levels of *Gb’cry2* mRNA were knocked down to nearly the basal level of that of intact crickets; although a small but significant fluctuation was observed (ANOVA: F_5,19_ = 5.44, *P* < 0.05 for ds*Gb’cry2#d1*; F_5,23_ = 3.18, *P* < 0.05 for ds*Gb’cry2#d2*, Fig. 2b), no significant daily rhythm was detected on single cosinor analysis (*P* > 0.05).

### Effects of *Gb’cry1* and *Gb’cry2* RNAi on the locomotor rhythm

**Fig. 3:**
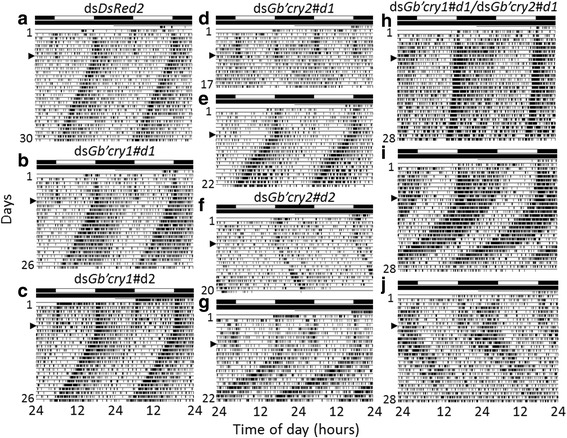
Double-plotted actograms showing locomotor rhythms of crickets *Gryllus bimaculatus*. (**a**–**j**) Locomotor activity of *DsRed2* ^RNAi^ (**a**), *Gb’cry1* ^RNAi^ (**b**, **c**), *Gb’cry2* ^RNAi^ (**d**–**g**), or *Gb’cry1* ^RNAi^ and *Gb’cry2* ^RNAi^ (**h**–**j**) crickets recorded under LD 12:12 and DD at a constant temperature of 25 °C. *Arrowheads* indicate the day when the crickets were transferred from LD to DD. *Gb’cry1* ^RNAi^ crickets showed a locomotor rhythm free-running in DD with a period similar to that of *DsRed2* ^RNAi^ crickets (**a**–**c**), but *Gb’cry2* ^RNAi^ and *Gb’cry1*/*Gb’cry2* double RNAi crickets showed variable free-running periods (**d**–**j**). For further explanations, see the text

**Fig. 4:**
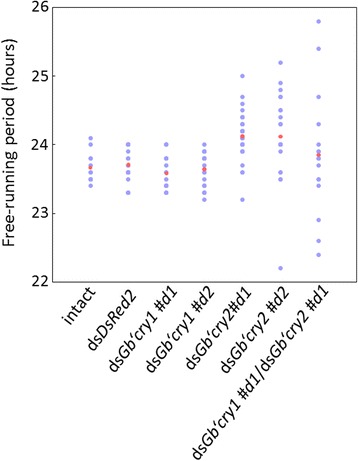
Distribution of free-running periods of locomotor rhythms of individual crickets *Gryllus bimaculatus*. Crickets were treated with *DsRed2* ^RNAi^, *Gb’cry1* ^RNAi^ (ds*Gb’cry1#d1*, ds*Gb’cry1#d2*), *Gb’cry2* ^RNAi^ (ds*Gb’cry2#d1*, ds*Gb’cry2#d2*), or both *Gb’cry1* ^RNAi^ and *Gb’cry2* ^RNAi^ and their locomotor rhythms were measured under DD at a constant temperature of 25 °C. *Blue* and *red dots* indicate individual and average values, respectively. *Gb’cry1* ^RNAi^ crickets showed a similar distribution to that of control, *DsRed2* ^RNAi^ crickets. The distribution is widely ranging from 23.2 to 25.0 h in *Gb’cry2* ^RNAi^ crickets, and the range is even greater in *Gb’cry1*/*Gb’cry2* double RNAi crickets. For further explanations, see the text

To examine the role of *Gb’cry1* and *Gb’cry2* in circadian locomotor rhythm regulation, locomotor activity was recorded in adult males injected with ds*Gb’cry1* (ds*Gb’cry1#d1*, *N* = 19; ds*Gb’cry1#d2, N* = 22) or ds*Gb’cry2* (ds*Gb’cry2#d1*, *N* = 37; ds*Gb’cry2#d2*, *N* = 22). Since similar results were obtained in crickets treated with two different dsRNAs for *Gb’cry1* and *Gb’cry2*, the results were pooled. We used *DsRed2* ^RNAi^ crickets (*n* = 21) as a negative control. As was the case for *DsRed2* ^RNAi^ crickets, all of the *Gb’cry1* ^RNAi^ and the *Gb’cry2* ^RNAi^ crickets exhibited a nocturnal activity rhythm under LD12:12, with a major peak at lights-off and a minor peak at lights-on (Fig. 3). In the ensuing constant darkness (DD), the rhythm free-ran, except in 4 *Gb’cry2* ^RNAi^ crickets which became arrhythmic, and the free-running periods varied with treatments (Figs. 3 and 4). The *Gb’cry1* ^RNAi^ (*n* = 16) and *DsRed2* ^RNAi^ (*n* = 15) crickets showed free-running periods shorter than 24 h, averaging 23.58 ± 0.25 h and 23.71 ± 0.24 h, respectively. However, the free-running period varied widely in *Gb’cry2* ^RNAi^ crickets (*n* = 33): the average period was 24.12 ± 0.34 h ranging from 23.2 h to 25.0 h. When treated doubly with ds*Gb’cry1* and ds*Gb’cry2,* the crickets were all rhythmic but showed a wider range of free-running period (22.4–25.8 h) than *Gb’cry2* ^RNAi^ crickets. Notably, some of the *Gb’cry2* ^RNAi^ crickets (ds*Gb’cry2#d1*, *n* = 3; ds*Gb’cry2#d2*, *n* = 2) showed a rhythm that split into two components in the free-running condition (as exemplified in Fig. 3d) and that individuals with the free-running periods longer than 24 h often became arrhythmic (ds*Gb’cry2#d1*, *N* = 4) or only weakly rhythmic (ds*Gb’cry2#d1*, *n* = 8; ds*Gb’cry2#d2*, *n* = 10) in due course (as exemplified in Fig. 3d, j).

### Effects of *Gb’cry1* and *Gb’cry2* RNAi on the molecular clockwork

**Fig. 5:**
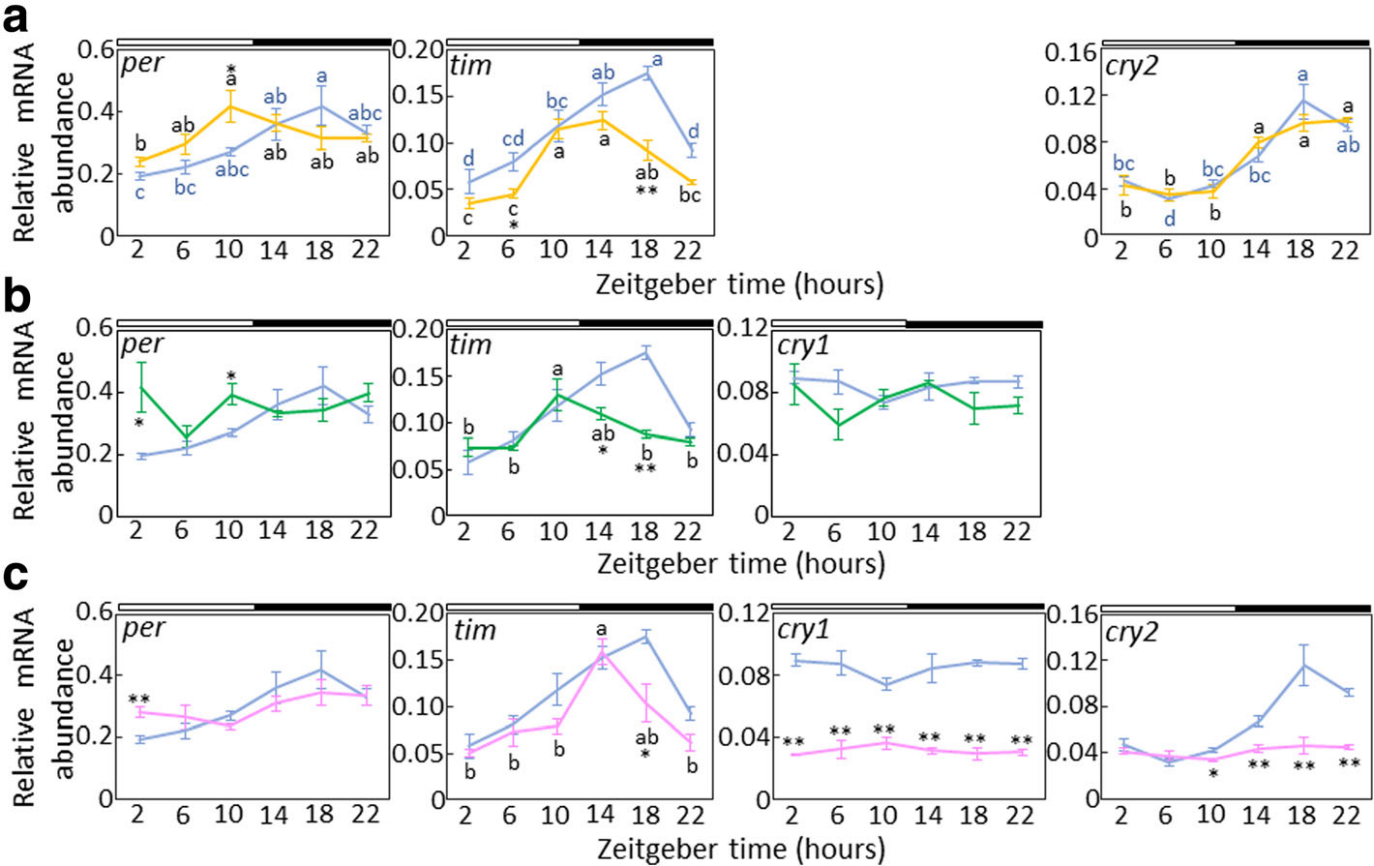
Effects of *Gb’cry1* ^RNAi^ (**a**), *Gb’cry2* ^RNAi^ (**b**), and *Gb’cry1* ^RNAi^/*Gb’cry2* ^RNAi^ (**c**) on daily clock gene expression. Relative abundance of *Gb’per*, *Gb’tim*, *Gb’cry1*, and *Gb’cry2* mRNA in the optic lobes are shown for intact (*blue*) and *Gb’cry1* ^RNAi^ (*yellow*) (**a**), *Gb’cry2* ^RNAi^ (*green*) (**b**), and *Gb’cry1* ^RNAi^/*Gb’cry2* ^RNAi^ (*pink*) adult male crickets (*Gryllus bimaculatus*) (**c**). The data for intact *Gb’cry1* and *Gb’cry2* mRNA are replotted from Fig. 2. In dsRNA-injected crickets, the optic lobes were collected about seven days after the dsRNA injection. The abundance of mRNA was measured by quantitative real-time RT-PCR with total RNA extracted from the optic lobes. The data collected from 3 and 5 independent experiments were averaged and plotted as mean ± SEM for dsRNA-injected and intact crickets, respectively. The abundance of *Gb’rpl18a* mRNA was used as an internal reference. Asterisks indicate significant difference between intact and treated crickets (*P < 0.05, **P < 0.01, *t-*test). Different letters indicate that values are significantly different with each other (ANOVA followed by Tukey-test, *P* < 0.05). For further explanations, see the text

We then examined the effects of RNAi of *Gb’cry1* and *Gb’cry2* on *Gb’per* and *Gb’tim* genes, using ds*Gb’cry1#d1* and ds*Gb’cry2#d1*. In intact crickets, transcripts of *Gb’per* and *Gb’tim* showed a rhythmic expression to peak around midnight (ZT18). These expression profiles were similar to those reported previously [9–11]. When *Gb’cry1* was knocked-down by RNAi, *Gb’cry2* and *Gb’tim* maintained a rhythmic expression (ANOVA, F_5,24_ = 15.10, *P* < 0.01; cosinor, *P* < 0.05 for *Gb’cry2* and ANOVA, F_5,23_ = 21.38, *P* < 0.01; cosinor, *P* < 0.05 for *Gb’tim*) with a peak similar to that of untreated crickets (*Gb’cry2*) or advanced by about 4–8 h to peak during late day to early night (*Gb’tim*); *Gb’per* also showed a similar rhythmic pattern to *Gb’tim*, showing a significant fluctuation (ANOVA, F_5,21_ = 3.59, *P* < 0.05), but no daily rhythm was detected by the single cosinor method (*P* > 0.05) (Fig. 5a). The results of *Gb’cry2* RNAi are shown in Fig. 5b. No clear effect was observed on *Gb’cry1* mRNA levels. *Gb’tim* showed a rhythm (ANOVA, F_5,20_ = 3.90, *P* < 0.05; cosinor, *P* < 0.05) with a peak at late day (ZT10), while *Gb’per* fluctuated around a medium range of the control, with no significant daily rhythm (ANOVA, F_5,21_ = 1.54, *P* > 0.23; cosinor, *P* > 0.05).

When *Gb’cry1* and *Gb’cry2* were simultaneously knocked down, both *Gb’cry1* and *Gb’cry2* showed no significant daily rhythm (Fig. 5c: ANOVA, F_5,22_ = 0.54, *P* > 0.73; cosinor, *P* > 0.05 for *Gb’cry1* and ANOVA, F_5,22_ = 2.14 *P* > 0.11; cosinor, *P* > 0.05 for *Gb’cry2*). *Gb’per* showed an arrhythmic expression pattern with mRNA levels around a medium range of control (ANOVA, F_5,23_ = 2.02, *P* > 0.12; cosinor, *P* > 0.05), while *Gb’tim* maintained a robust oscillation with a peak slightly advanced to peak at ZT14 (Fig. 5c: ANOVA, F_5,20_ = 9.36, *P* < 0.01; cosinor, *P* < 0.05).

### *Gb’cry1* and *Gb'cry2* regulates CLK/CYC transcriptional activity

**Fig. 6:**
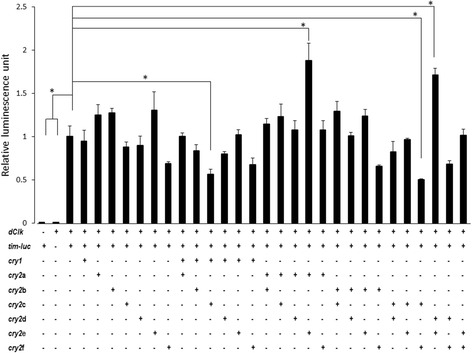
Suppressive effects of *Gb’*CRY1 or *Gb’*CRY2 proteins on *tim* transcription. Relative luciferase activity of *tim-luc* was measured in the presence of 100 ng of *pAc-Clk* with various combinations of the indicated *pAc5.1B-Gb’cry1, pAc5.1B-Gb’cry2*a, -b, -c, -d, -e, or -f expression plasmids. Signals were normalized to *Rluc* activity and then to the control signal of cells transfected with 100 ng of *tim-luc* and *pAc-Clk*. Error bars represent SEM (*n* = 3). A combination of *Gb’cry2*b and *Gb’cry2*c suppressed the transcription of E-box genes. *Asterisks* represents *P* < 0.01 versus control (Dunnett’s multiple range test)

We examined the role of * Gb'cry1* and *Gb’cry2* in the molecular clockwork using cell-based assay. Various combinations of *Gb’cry1* and *Gb’cry2*a ~ f were transfected into S2 cells together with *Drosophila Clk* and *Drosophila tim*-*Luc*. When *Gb’cry2*c was co-transfected with *Gb’cry1* or *Gb’cry2*f, transcriptional activity of CLK/CYC was significantly suppressed (Dunnett’s test, *P* < 0.05; Fig. 6). CLK/CYC transcriptional activity was enhanced when *Gb’cry2*e was co-transfected with *Gb’cry2*a or *Gb’cry2*d (Dunnett’s test, *P* < 0.05; Fig. 6). Transfection in other combination had no significant effects on transcriptional activity through the E-box.

### Effects of RNAi of *Gb’per*, *Gb’tim*, *Gb’Clk* and *Gb’cyc* on *Gb’cry1* and *Gb’cry2* expression

**Fig. 7:**
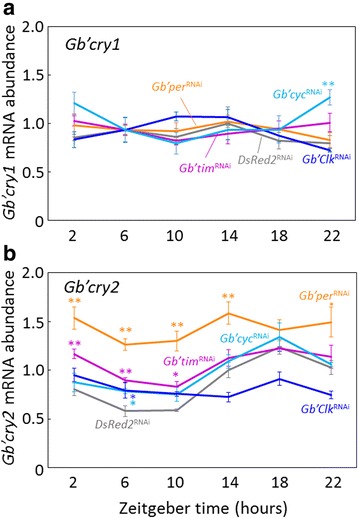
Effects of RNAi of various clock genes on *Gb’cry1* **a** and *Gb’cry2* mRNA **b** in the cricket optic lobes. Adult male crickets (*Gryllus bimaculatus*) were treated with *DsRed2* ^RNAi^ (*gray*), *Gb’per* ^RNAi^ (*orange*), *Gb’tim* ^RNAi^ (*purple*), *Gb’Clk* ^RNAi^ (*blue*), and *Gb’cyc* ^RNAi^ (*light blue*). The optic lobes were collected about 7 days after the dsRNA injection. The abundance of mRNA was measured by quantitative real-time RT-PCR with total RNA extracted from the optic lobes. The data collected from three and four independent experiments were averaged and plotted as mean ± SEM for clock gene dsRNA-injected and ds*DsRed2*-injected crickets, respectively. The abundance of *Gb’rpl18a* mRNA was used as an internal reference. The data for *DsRed2* ^RNAi^ are replotted from Fig. 2. Asterisks indicate significant difference between *DsRed2* ^RNAi^ crickets and those treated with clock gene dsRNAs: *P < 0.05, **P < 0.01, Dunnett’s test. Note that *Gb’cry1* mRNA levels were never affected by RNAi of clock genes except for *cyc* ^RNAi^ at ZT22, and that *Gb’cry2* mRNA levels showed a rhythmic expression after *Gb’tim* or *Gb’cyc* dsRNA treatments, but became arrhythmic when treated with *Gb’per* or *Gb’Clk* dsRNA. For further explanations, see the text

To investigate the relationship of *Gb’cry1* and *Gb’cry2* with a *Gb’per/Gb’tim* loop, we examined the effects of RNAi of *Gb’per*, *Gb’tim*, *Gb’Clk* and *Gb’cyc* on the expression levels of *Gb’cry1* and *Gb’cry2*. These treatments have shown that molecular oscillation of respective gene is eliminated [9–12]. The RNAi of these clock genes had no clear effects on *Gb’cry1* mRNA levels except for *Gb’cyc* ^RNAi^ at ZT22, at which a significant increase in *Gb’cry1* mRNA was observed (Fig. 7a). The same treatments, however, significantly affected *Gb’cry2* mRNA expression (Fig. 7b). *Gb’per* ^RNAi^ upregulated the *Gb’cry2* mRNA levels to raise the bottom level to exceed the peak level of control crickets and lead to arrhythmic expression (ANOVA, F_5,18_ = 1.12, *P* > 0.39; cosinor, *P* > 0.05). In *Gb’Clk* ^RNAi^ crickets, *Gb’cry2* mRNA showed no expression rhythm, remaining at the median range (ANOVA, F_5,17_ = 2.01, *P* > 0.14; cosinor, *P* > 0.05). *Gb’cry2* mRNA retained a daily rhythm with a normal level in *Gb’tim* ^RNAi^ and *Gb’cyc* ^RNAi^ crickets (ANOVA, F_5,23_ = 4.62, *P* < 0.01; cosinor, *P* < 0.05 for *Gb’tim* ^RNAi^ and ANOVA, F_5,17_ = 7.02, *P* < 0.01; cosinor *P* < 0.05 for *Gb’cyc* ^RNAi^), but the amplitude was slightly attenuated with the increased levels during daytime. These results strongly suggest that rhythmic expression of *Gb’cry2* is regulated by a mechanism other than the *Gb’per/Gb’tim* loop.

## Discussion

### *Gb’cry1* and *Gb’cry2* genes of the cricket

In insects, two *cry* genes have been identified: one is the *Drosophila*-type *cry1* and the other is the mammalian-type *cry2* [3, 22]. The present study showed that the cricket, *Gryllus bimaculatus* also has two *cry* genes. *Gb’cry2* has six splicing variants with variations in exons 2, 4, 6 and 10. The expression levels were different among those variants and greatest in *Gb’cry2*f with an exon 4, long exons 2 and 6, and a short exon 10. Their daily expression profiles in the optic lobe are similar to those in other insects; *Gb’cry2* showed a rhythmic expression with a peak around mid-night, while *Gb’cry1* is rather constitutively expressed throughout a day with a slight reduction at the late day phase. The reduction of *Gb’cry1* may be caused by a light-induced suppression mechanism that is known to regulate *cry1* in other insects [3]. *Gb’cry1* expression is most likely independent of the circadian clock, since RNAi of clock genes had nearly no effect on *Gb’cry1* mRNA levels (Fig. 7a).

### Role of *Gb’cry1* and *Gb’cry2* in the cricket’s clock

In flies and butterflies, CRY1 is supposed to be a blue light receptor, and resets the clock by leading to a degradation of TIMELESS protein in a light-dependent manner [4, 6, 23]. Our results, however, clearly show that *Gb’cry1* ^RNAi^ crickets synchronized to the given light dark cycle as robustly as the control crickets, suggesting that *Gb’*CRY1 is not the major photoreceptor for entrainment of the clock. This finding is consistent with our previous reports that the compound eye is the only photoreceptor necessary for light entrainment of the cricket clock [24] and that opsin-long wavelength (*opsin-LW*) expressed in the compound eye plays a major role in photic entrainment [25]. Similarly *Gb’cry2* is apparently not a major photoreceptor for entrainment, because *Gb’cry2* ^RNAi^ crickets also robustly synchronized to light cycles, similar to the case for *Gb’cry1* ^RNAi^ crickets. However, we could not exclude the possibility that the two *cry* genes play subtle roles in photic entrainment. To draw a conclusion on the involvement of these genes in photic entrainment, careful examination of entrainability in *Gb’cry1* ^RNAi^ and *Gb’cry2* ^RNAi^ crickets is needed.

On the free-running rhythm in DD, *Gb’cry2* ^RNAi^ had significant effects; the treated crickets showed variable free-running periods, suggesting that *Gb’cry2* plays a significant role in the clock oscillatory mechanism. In contrast, *Gb’cry1* ^RNAi^ had no clear effect, and the treated crickets showed a free-running rhythm with a period similar to that of control crickets treated with ds*DsRed2*. However, *Gb’cry1* ^RNAi^ enhanced the variability of the free-running period of *Gb’cry2* ^RNAi^ crickets (Figs. 3, 4), suggesting that *Gb’cry1* and *Gb’cry2* cooperate to determine the free-running period.

### *Gb’*CRYs are transcriptional repressors

The present study showed that *Gb’*CRYs play in the cricket molecular clockwork as transcriptional repressors (Fig. 6). We have shown for the first time that *Gb’cry2* has six splicing variants and that none has a repressor activity alone. The repressor activity was evident only when *Gb’cry2*c was co-expressed with *Gb’cry1* or *Gb’cry2*f (Fig. 6). The requirement of two types of CRYs contrasts to mammalian and other insect clocks where a single molecular species of CRY can work as transcriptional repressor [3, 26, 27]. Since *Gb’cry2*c, *Gb’cry2*f, and *Gb’cry1* commonly have ICAT and RD-2b, the former is likely important for repression of CLK/CYC transcriptional activity [28] and the latter may be required for the nuclear translocation [29]. *Gb’cry1*’s role as a transcriptional repressor in combination with *Gb’cry2* (Fig. 6) is reminiscent of a finding from peripheral tissues of *Drosophila*, in which *cry1* is also known to function as a transcriptional repressor [23, 30]. For example, overexpression of *cry1* and *per* in the *Drosophila* compound eye represses CLK/CYC-activated transcription [30]. However, the *Gb’cry1*’s role in transcriptional regulation is contrast to an earlier report on *Anopheles gambiae*, *Antheraea pernyi,* and *Danaus plexippus,* in which *cry1* has no repressor activity [3]. The role of *cry1* in the core clock mechanism may be lost in these insects, in which *cry2* plays a major role as a transcriptional repressor. However, we also suggest that the role of *cry1* should be carefully reexamined in those insects, since Yuan et al. [3] did not examine the role of *cry1* in combination with *cry2*.

The role of *Gb’*CRYs as a component in the clock machinery partly explains the changes of free-running periods in *Gb’cry2* ^RNAi^ crickets. Even after RNAi treatment it is expected that a small amount of mRNA will be expressed. The variable amount of expressed *Gb’cry2* probably resulted in variable free-running periods among individuals treated by *Gb’cry2* ^RNAi^ or *Gb’cry1*/*Gb’cry2* double RNAi (Fig. 4) as suggested for CRYs in the mammalian suprachiasmatic nucleus (SCN) clock [31]. In *Gb’cry1* ^RNAi^ crickets, the lack of *Gb’cry1* may be compensated by another complex formed by *Gb’*CRY2c and *Gb’*CRY2f, which would account for the lack of any observable significant changes in this period.

Our reporter assay also showed that CLK/CYC transcriptional activity was enhanced when *Gb’cry2*e was co-expressed with *Gb’cry2*a or *Gb’cry2*d. Although this possibility cannot be ruled out, their role as transcriptional activator may be negligible, since *Gb’cry2*e was expressed at only trace levels and the expression of *Gb’cry2*a and *Gb’cry2*d was also quite low (Fig. 2c).

Another possible role of *Gb’cry*2 may be as a coupling factor between clock neurons. This hypothesis is based on the observation that *Gb’cry2* ^RNAi^ crickets sometimes show a rhythm dissociation into some components running with different free-running periods in DD (Fig. 3d). The change in coupling strength by *Gb’cry2* ^RNAi^ may affect in variable degrees the free-running period of the locomotor rhythm. This may represent a parallel case with that described in reports on mammalian clocks in the SCN [32, 33]. Some of the *Gb’*CRY2 variants might play a role in this coupling and/or period change in individual clock neurons caused by *Gb’cry2* ^RNAi^ may indirectly affect the coupling. This issue remains to be addressed in future studies.

### *Gb’cry2* oscillation is regulated by a mechanism different from the *Gb’per/Gb’tim* loop

**Fig. 8:**
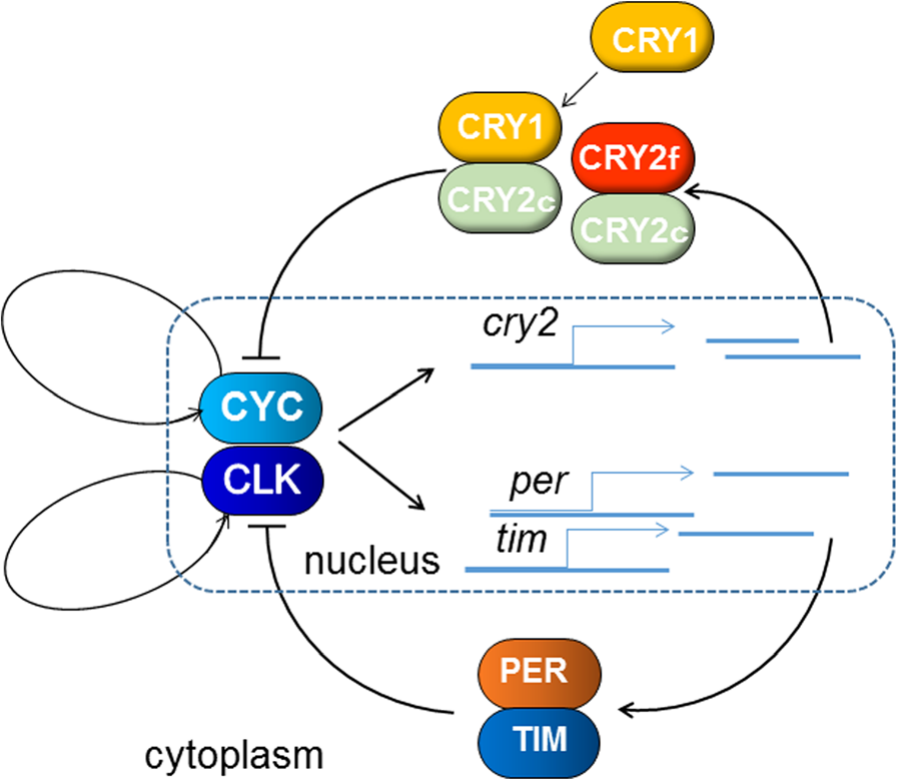
A model for the cricket circadian clock. The clock includes two negative feedback loops, one for *Gb’per*/*Gb’tim* like in *Drosophila* and the other for *Gb’cry2*. The latter is comprised of two *Gb’cry2* variants, *Gb’cry2*c and *Gb’cry2*f, or *Gb’cry2*c and *Gb’cry1*, and their product proteins that form a complex to suppress the transcription mediated by *Gb’*CLK/*Gb’*CYC complex. These two loops may be able to operate independently but interact with each other through influence on the *Gb’*CLK/*Gb’*CYC. In addition, there are two other loops for rhythmic expression of *Gb’Clk* and *Gb’cyc* [12]

We have shown that the cricket circadian clock includes a loop for rhythmic expression of *Gb’per* and *Gb’tim* [9, 10], of which transcription is regulated by *Gb’*CLK/*Gb’*CYC [11, 12]. It has been suggested that CRY2 forms a complex with PER or the PER/TIM complex to enter the nucleus and repress the CLK/CYC transcriptional activity in other insects [5, 6]. However, the results of the present study suggest that *Gb’*CRY2 composes a loop to repress *Gb’*CLK/*Gb’*CYC activity by forming complex between its variants or with *Gb’*CRY1, and that the loop can operate independent of the *Gb’per/Gb’tim* loop to some degree (Fig. 8). This hypothesis is based on the following observations. When *Gb’tim* was knocked down by RNAi or *Gb’tim* and *Gb’per* stopped their oscillation by *Gb’cyc* ^RNAi^ [12], *Gb’cry2* maintains its oscillation within a normal range but with a slightly attenuated amplitude (Fig. 7). Alternatively when *Gb’cry2* is knocked down, *Gb’tim* maintained its oscillation. Our data suggest that the circadian locomotor rhythm is expressed when either *Gb’cry2* or *Gb’tim* is rhythmically expressed.

However, there must be an inter-connection between the assumed *Gb’cry2* loop and the *Gb’per/Gb’tim* loop. When *Gb’per* or *Gb’Clk* was knocked down by RNAi, *Gb’cry2* lost its rhythmic expression (Fig. 7), and in *Gb’per* ^RNAi^ crickets the mRNA levels of *Gb’cry2* were significantly increased, with the trough level nearly equivalent to the peak level of *DsRed2* ^RNAi^ crickets (Fig. 7). Alternatively when *Gb’cry2* was knocked down, *Gb’per* lost the rhythm but *Gb’tim* showed a phase advance of its expression rhythm (Fig. 5). Although further detailed study is necessary to understand the underlying mechanism, these data suggest that some coupling mechanism exists between the two loops. For *Gb’per*, *Gb’tim* and *Gb’cry2*, *Gb’*CLK/*Gb’*CYC may be a common transcriptional activator. Thus, the two loops may couple with *Gb’*CLK/*Gb’*CYC as a hinge (Fig. 8). The transcriptional activity of *Gb’*CLK/*Gb’*CYC may be suppressed by both *Gb’*PER/*Gb’*TIM complex and *Gb’*CRY1/*Gb’*CRY2 or *Gb’*CRY2 complexes. Knock-down of either *Gb’cry1* or *Gb’cry2* weakens the repression of CLK/CYC, resulting in the upregulation of *Gb’per* mRNA. The transcription of *Gb’tim* and *Gb’cry2* may require stronger transactivation than *Gb’per,* and thus *Gb’tim* and *Gb’cry2* mRNA maintained rhythmic expression even after knockdown of *Gb’cry1/Gb’cry2* and *Gb’tim*, respectively.

## Conclusion

The cricket clock consists of at least four loops, each of which regulates rhythmic expression of *Gb’per* and *Gb’tim*, *Gb’Clk*, *Gb’cyc*, and *Gb’cry2* [9, 10, 12] (Fig. 8). The clock system appears to be more complex than those of *Drosophila* and other insects, which basically include three loops for rhythmic expression of *per* and *tim*, *Clk* or *cyc*, and *clockwork orange* [1, 34]. The cricket is a hemimetabolous insect, and therefore more primitive than holometabolous insects, including Diptera (*Drosophila*), Lepidoptera (*Danaus*, *Antheraea*), and Hymenoptera (*Apis*). It may thus possess a more primitive clock machinery, of which some components may have changed or been lost in the course of phylogenetic development in higher insects.

The cricket’s clock possesses some properties common to mammalian clock. *Gb’cyc* shows a rhythmic expression similar to that of its mammalian homologue, *Bmal1* [12]. Both *Gb’cry1* and *Gb’cry2* are involved in the clock machinery. The reset mechanism is exclusively relies on retinal photoreceptors [24, 25, 35]. Similarly the firebrat *Thermobia domestica*, one of the most primitive ametabolous insects, has a mammalian-like clock machinery in which *cyc* is rhythmically expressed [36, 37]. These findings suggest that the ancestral insect clock resembled the mammalian clock and subsequently diversified into various types [1].

The evolution and diversification of insect *cry* genes remains a challenging issue. There is a variation for presence and absence of *cry* genes. Although many insects have both *cry1* and *cry2*, some dipteran species lack *cry2* and some coleopteran and hymenopteran species lack *cry1* [1, 38]. The present study revealed that *Gb’*CRY1 is not a photoreceptor, but rather a component of the clock and that *Gb’cry2* has splicing variants, providing another example of diversification of *cry1* and *cry2*. These findings make it difficult to resolve the evolution or diversification of insect *cry* genes. To deal with this issue, extensive comparative studies are desired across phylogenetically diverse species, including hemimetabola and ametabola.

## Abbreviations

ANOVA: Analysis of variance
*Clk*: *Clock*
CLK: CLOCK
*cry*: *cryptochrome*
CRY: CRYPTOCHROME
*cry1*: *cryptochrome 1*
CRY1: CRYPTOCHROME 1
*cry2*: *cryptochrome 2*
CRY2: CRYPTOCHROME 2
*cyc*: *cycle*
CYC: CYCLE
DD: Constant darkness
dsRNA: Double stranded RNA
*Gb*: *Gryllus bimaculatus*
ICAT: Inhibition of CLOCK-ARNTL-mediated transcription domain
LD: Light/dark
*opsin-LW*: *Opsin-long wavelength*
*per*: *period*
PER: PERIOD
qPCR: Quantitative real-time reverse transcription polymerase chain reaction
RACE: Rapid amplification of cDNA ends
RNAi: RNA interference
*rpl18a*: *ribosomal protein L18a*
RT-PCR: Reverse transcription polymerase chain reaction
SCN: Suprachiasmatic nucleus
*tim*: *timeless*
TIM: TIMELESS
ZT: Zeitgeber time
